# Chromatin-Remodelling ATPases *ISWI* and *BRM* Are Essential for Reproduction in the Destructive Pest *Tuta absoluta*

**DOI:** 10.3390/ijms23063267

**Published:** 2022-03-17

**Authors:** Shun-Xia Ji, Qiang-Wen Wu, Si-Yan Bi, Xiao-Di Wang, Gui-Fen Zhang, Fang-Hao Wan, Zhi-Chuang Lü, Wan-Xue Liu

**Affiliations:** 1 State Key Laboratory for Biology of Plant Diseases and Insect Pests, Institute of Plant Protection, Chinese Academy of Agricultural Sciences, Beijing 100193, China; 82101172334@caas.cn (S.-X.J.); wu210375@email.swu.edu.cn (Q.-W.W.); bisiyan91@gmail.com (S.-Y.B.); 82101195160@caas.cn (X.-D.W.); zhangguifen@caas.cn (G.-F.Z.); wanfanghao@caas.cn (F.-H.W.); liuwanxue@caas.cn (W.-X.L.); 2 Agricultural Genome Institute at Shenzhen, Chinese Academy of Agricultural Sciences, Shenzhen 518120, China

**Keywords:** bromo, fecundity, imitation SWItch, RNA interference, *Tuta absoluta*

## Abstract

The tomato leaf miner (*Tuta absoluta*) is one of the top 20 plant pests worldwide. We cloned and identified the chromatin-remodelling ATPase genes *ISWI* and *BRM* by RACE and bioinformatic analysis, respectively; used RT-qPCR to examine their expression patterns during different life cycle stages; and elucidated their roles in insect reproduction using double-stranded RNA injections. The full-length cDNA of *TaISWI* was 3428 bp and it encoded a 1025-aa polypeptide. The partial-length cDNA of *TaBRM* was 3457 bp and it encoded a 1030-aa polypeptide. *TaISWI* and *TaBRM* were upregulated at the egg stage. Injection of *TaISWI* or *TaBRM* dsRNA at the late pupa stage significantly inhibited adult ovary development and reduced fecundity, hatchability, and longevity in the adult females. To the best of our knowledge, the present study was the first to perform molecular characterisations of two chromatin-remodelling ATPase genes and clarify their roles in *T. absoluta* fecundity. Chromatin-remodelling ATPases are potential RNAi targets for the control of *T. absoluta* and other insect pests. The present study was also the first to demonstrate the feasibility of reproductive inhibitory RNAi as a putative approach for the suppression of *T. absoluta* and other Lepidopteran insect populations.

## 1. Introduction

The South American tomato leaf miner *Tuta absoluta* (Meyrick) (Lepidoptera: Gelechiidae) is an invasive, destructive pest originating from Peru [1]. It has been one of the worst pests of tomato in South America since the 1950s [2,3]. It was detected in eastern Spain in 2006 and continued to spread across most countries of Europe, Africa, the Middle East, and Asia [4,5,6,7]. To date, *T. absoluta* has been detected and recorded in over 90 countries and regions worldwide [8]. A recent estimate indicated that *T. absoluta* could continue to expand its range by 800 km/y [9]. *T. absoluta* was detected in Ili, Xinjiang Province, China, in August 2017 [7]. This region is a major global tomato producer [10]. Over the following two years, *T. absoluta* was detected at 269 sites in 33 prefectures, one county-level city in Chongqing, and seven provinces in northwest and southwest China. *T. absoluta* colonised ≈11,635.8 km (48.5%) of roadway across the Chinese mainland [11]. Hence, this insect pest has invaded broadly and rapidly. *T. absoluta* infests the leaves, flowers, stems, and fruits of solanaceous crops such as potato, eggplant, bell pepper, tobacco, and especially tomato, and can lower crop yield by 80–100% [7,12,13]. *T. absoluta* has become a serious threat to global greenhouse and open-field tomato production [4,9,14].

Synthetic insecticides are extensively used to control tomato leaf miner. However, prolonged, frequent chemical pesticide application has resulted in overuse in many tomato planting areas. This management practice has disrupted integrated pest control strategies employing natural enemy insects [15,16]. *T. absoluta* has high reproductive potential (260–350 eggs/female) and a short generation time (10–12/y) [4,17]. Hence, it could rapidly acquire resistance to multiple pest control products [18,19,20,21]. Compared with a susceptible reference strain, chlorantraniliprole and flubendiamide (diamide) resistance were 2414-fold and 1742-fold higher, respectively, in Italian *T. absoluta* populations [19]. Therefore, novel, efficacious control strategies against *T. absoluta* are urgently needed [22,23,24].

RNA interference (RNAi) is an evolutionarily conserved nucleic acid metabolism mechanism that can be initiated by exogenous application or endogenous expression of double-stranded RNAs (dsRNA). It is a powerful tool for functional genomic studies and has great potential in agricultural pest management [25,26,27]. When the sequences of dsRNA or small interfering RNA (siRNA) matched those of vital target insect genes, the latter were silenced. Consequently, growth and/or development and/or reproduction were impaired, and death sometimes occurred in the pest [28]. Given the great potential of RNAi strategies, they will be widely used in the control of agricultural pests in fields in the future. Therefore, potential gene targets must be identified to establish an efficient and robust RNAi system for pest control [29,30].

ATP-dependent chromatin-remodelling factors are multi-subunit protein complexes. They utilise energy from ATP hydrolysis to modulate the access of transcription factors (TFs) and other regulatory proteins to genomic DNA that regulate target gene expression [31,32,33]. These factors were divided into four different families on the basis of their conserved domains: ISWI (Imitation SWItch), SWI/SNF (SWItch/Sucrose Non-Fermentable), INO80 (INOsitol requiring 80), and CHD (Chromo Helicase Domain) [34]. Recent studies showed that chromatin-remodelling genes play important regulatory roles in insect development and reproduction. Khajuria et al. (2015) [35] observed that total oviposition was not significantly affected in female western corn rootworms (*Diabrotica virgifera virgifera*) exposed to the dsRNA of brm encoding SWI/SNF complex ATPases. Nevertheless, this treatment fully inhibited egg hatching. Fishilevich et al. (2016) [36] showed that dsRNA targeting the chromatin-remodelling ATPase transcripts brm and iswi encoding the ISWI complex ATPases, and mi-2 encoding the CHD complex ATPases strongly reduced fecundity in western corn rootworm and Neotropical brown stink bug *(Euschistus heros*) females. In addition, knockdown of iswi, brm, and ino80 encoding the INO80 complex ATPases significantly reduced reproductive fitness and lifespan in the common bed bug *Cimex lectularius* [37,38].

It was commonly thought that chromatin-remodelling ATPases were associated with insect reproduction. Nevertheless, experimental evidence for this relationship in Lepidoptera pests is scarce. To the best of our knowledge, the present study is the first to investigate the functions of ATPase in the reproduction of the Lepidopteran pest *T. absoluta*. The chromatin-remodelling ATPases *ISWI* and *BRM* were cloned and identified by RACE and bioinformatic analysis. Their expression patterns at various life cycle stages were examined by qPCR. Their roles in insect reproduction were elucidated using double-stranded RNA (dsRNA) injection. It is believed that the findings of this research provide promising RNAi targets for the control of *T. absoluta.*

## 2. Results

### 2.1. Chromatin-Remodelling Gene Cloning

Full-length cDNA of *T. absoluta* ISWI was 3428 bp in length and was comprised of a 66-bp 5′-untranslated region (UTR) (positions 1–66), a 287-bp 3′-UTR (positions 3142–3428), and a 3075-bp open reading frame (ORF) (positions 67–3141). The ORF encoded a 1025-aa polypeptide with calculated MW = 118.83 kDa and isoelectric point = 7.81 (Figure S1A). The accession number of *T. absoluta* ISWI is OM937128.

The partial-length *T. absoluta* BRM cDNA was 3457 bp and it comprised a 63-bp 5′-UTR (positions 1–63), a 304-bp 3′-UTR (positions 3154–3457), and a 3090-bp ORF (positions 64–3153). The ORF encoded a 1030-aa polypeptide with calculated MW = 119.80 kDa and isoelectric point = 5.75 (Figure S1B). The accession number of *T. absoluta* BRM is OM937129.

### 2.2. TaISWI and TaBRM Characterisation

A conserved domains analysis showed that *TaISWI* contained the *N*-terminal ATPase domain (DEXDc: residues 127–318; HELICc: residues 468–552) and the *C*-terminal tandem HAND, SANT (SWI3, ADA2, N-CoR, and TFIIIB), and SLIDE (SANT-like ISWI) domains (residues 701–802, 803–844, and 859–971, respectively) (Figure S2A). *TaBRM* contained the BRK (Brahma and Kismet domain, residues 83–123), ATPase (DEXDc: residues 204–293), HELICc (residues 562–645), SnAC (SNF2 ATP-coupling domain, residues 748–808), and bromo (residues 864–964) domains (Figure S2B).

We also used I-TASSER to predict the 3D structures of the chromatin-remodelling ATPase genes. The DEXDc domain is shown in golden yellow, HELICc domain in pink, HAND domain in cyan, SANT domain in red, and SLIDE domain in blue (Figure S3A). Moreover, the BRK domain is shown in pink, DEXDc domain in golden yellow, HELICc domain in red, SnAC domain in brown, and bromo domain in purple (Figure S3B). Both of them had representative domains of the chromatin remodeller catalytic subunits.

**Figure 1:**
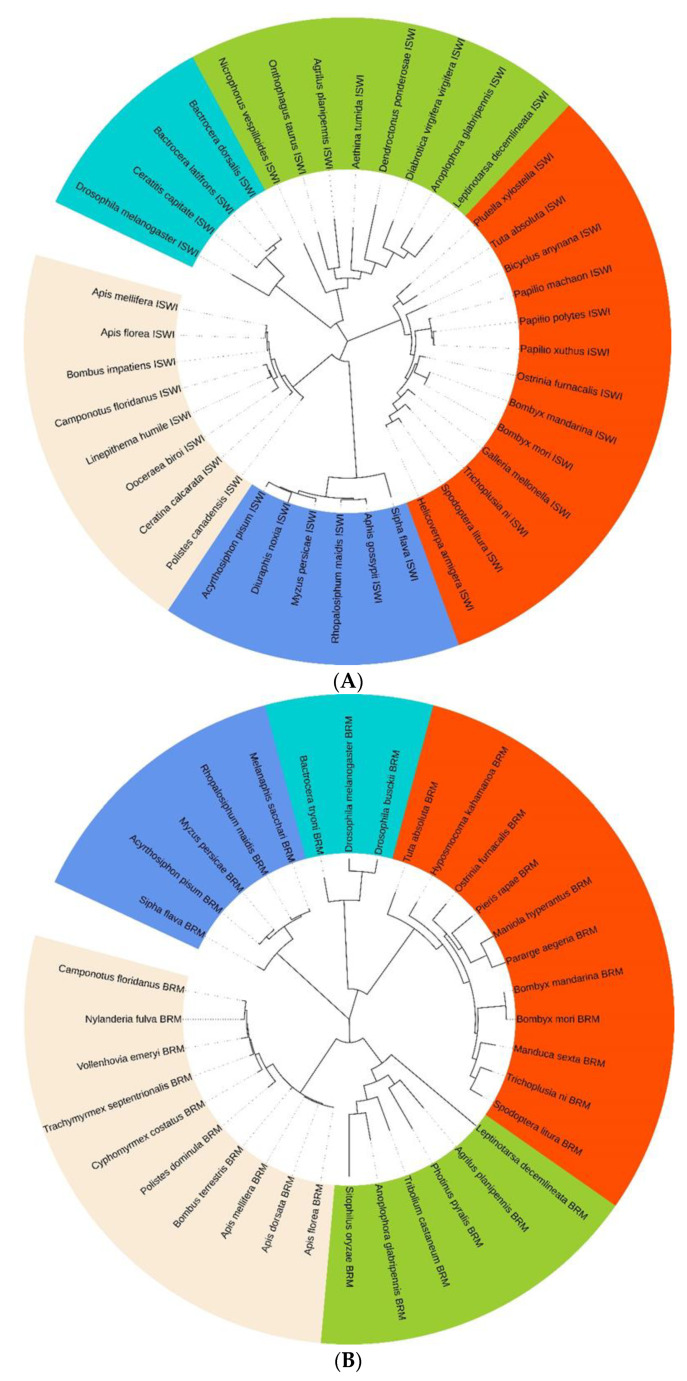
Phylogenetic tree based on known ISWI (**A**) and BRM (**B**) aa sequences. It was constructed in MEGA7.0 using ML method based on the Whelan Goldman (WAG) model with 1000 bootstrap replications. Lepidopterais depicted in vermilion; Hymenopterain in antique white; Coleopterain in yellow green; Diptera in sky blue; Hemipterain in cornflower blue. Table S1 lists ISWI and BRM protein sequence accession numbers.

A blastp analysis revealed that the deduced TaISWI amino acid sequence shared >82% identity with ISWI proteins previously identified in 65 other species. TaISWI had 93.55% similarity with the ISWI protein of the Lepidopteran *Helicoverpa armigera*. TaBRM had >72.01% homology with BRM proteins previously identified in 45 other species. TaBRM had 90.60% similarity with the BRM protein of the Lepidopteran *Trichoplusia ni*. We selected four or five representative species for multiple sequence alignment. The deduced TaISWI and TaBRM protein sequences were conserved relative to those previously identified for other insect species (Figure S4). Moreover, the phylogenetic analysis revealed that the various ISWI and BRM proteins clustered on single branches corresponding to each insect order (Figure 1). The foregoing results showed that both chromatin-remodelling ATPase genes were highly conserved across different insect species.

### 2.3. TaISWI and TaBRM Expression Patterns during Different Life Cycle Stages

**Figure 2:**
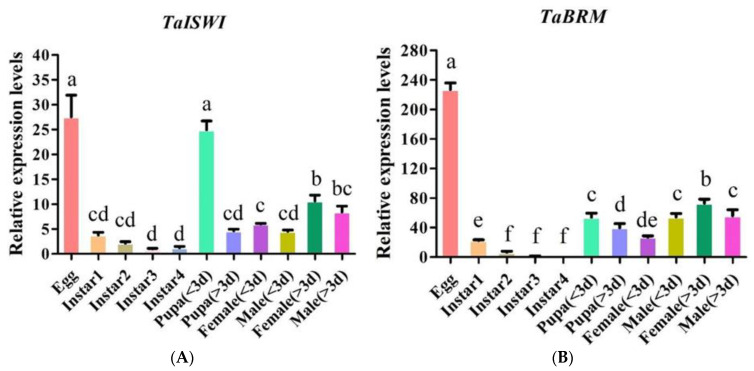
Relative to RpL5, *TaISWI* (**A**) and *TaBRM* (**B**) expression levels at different life cycle stages. Relative expression levels are means ± SEM. Bars with different lowercase letters indicate significant differences between treatment means at *p* < 0.05.

RT-qPCR showed that the *TaISWI* and *TaBRM* mRNAs were expressed at all tested developmental stages. Eggs (27.53 ± 7.59; *df* = 22; *p* < 0.05) and pupae < 3 d old presented with the highest *TaISWI* and *TaBRM* mRNA expression levels (24.93 ± 3.07; *df* = 22; *p* < 0.05) of all developmental stages. Peak *TaBRM* mRNA expression occurred in the eggs (227.02 ± 15.11; *df* = 22; *p* < 0.05). The expression level of both genes was significantly higher in the adults than the instars (Figure 2).

### 2.4. Effects of dsRNA Injection on TaISWI and TaBRM Expression

**Figure 3:**
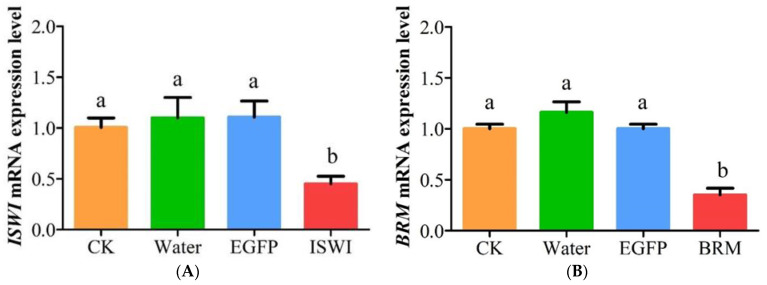
Effects of double-stranded RNA (dsRNA) treatment on *TaISWI* (**A**) and *TaBRM* (**B**) mRNA expression. Controls consisted of uninjected female pupae (CK) and those injected with water or *EGFP* dsRNA. Expression levels are means ± SEM. Bars with different lowercase letters indicate significant differences between treatment means at *p* < 0.05.

To elucidate the functions of the chromatin-remodelling genes, pre-emergent late female pupae were collected and injected with dsRNA to silence *TaISWI* and *TaBRM* expression. RT-qPCR demonstrated that the transcript levels of *TaISWI* ((45.39 ± 13.14)%; *df* = 8; *p* < 0.05) and *TaBRM* ((35.32 ± 11.35)%; *df* =8; *p* < 0.05) were significantly reduced in the female adults at 72 h after dsRNA injection (Figure 3).

### 2.5. Effects of dsRNA Injection on Ovarian Development and Fecundity

**Figure 4:**
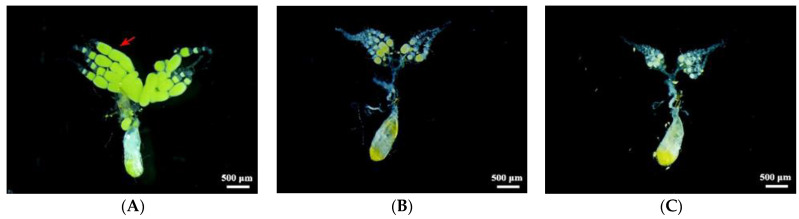
Effects of ds*ISWI* and ds*BRM* on *Tuta absoluta* ovary development. (**A**) Ovaries of uninjected 4 d females. (**B**) Abnormal ovaries of 4 d females injected with ds*ISWI*. (**C**) Abnormal ovaries of 4 d females injected with ds*BRM*. The red arrow represents the mature oocytes. The ovaries were photographed under an Olympus stereomicroscope (SZX16; Olympus, Tokyo, Japan). See more pictures in Figure S5.

Dissected ovaries revealed that there were significantly fewer mature oocytes in the 4 d females injected with ds*ISWI* or ds*BRM* than there were in the control insects (Figure 4).

**Figure 5:**
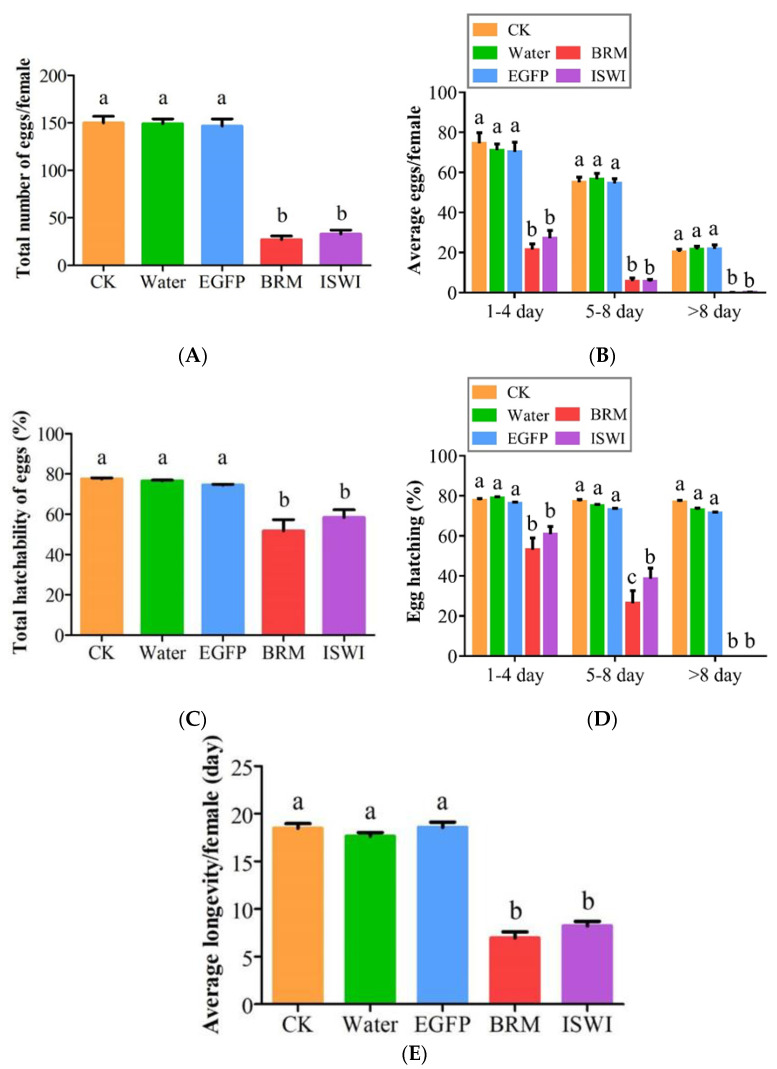
Effects of ds*ISWI* and ds*BRM* on *Tuta absoluta* oviposition, hatching, and longevity. (**A**) Average number of eggs laid per female at 1–4 d, 5–8 d, and >8 d. (**B**) Total oviposition per female. (**C**) Percentage of eggs hatched at 1–4 d, 5–8 d, and >8 d. (**D**) Total egg hatchability. (**E**) Average longevity of injected F0 females. Controls consisted of uninjected female pupae (CK) and those injected with water or *EGFP* dsRNA. Data are means ± SEM. Bars with different lowercase letters indicate significant differences between treatment means at *p* < 0.05.

Compared with the control, dsRNA *TaISWI* or *TaBRM* injection significantly reduced fecundity during the first 8 d and virtually stopped it altogether thereafter (Figure 5A). The total oviposition rates per female adult were 32.84 ± 18.36 (*df* = 72; *p* < 0.05) and 27.16 ± 16.36 (*df* = 72; *p* < 0.05) after *ISWI* and *BRM* silencing, respectively. The total number of eggs per uninjected female was 149.95 ± 30.25 (*df* = 72; *p* < 0.05). The total numbers of eggs per female were 149.16 ± 21.30 (*df* = 72, *p* < 0.05) and 146.63 ± 32.13 (*df* = 72; *p* < 0.05) following water and *EGFP* dsRNA injection, respectively (Figure 5B).

The hatching rates of the females were ((58.39 ± 16.68)%; *df* = 72; *p* < 0.05) and ((51.71 ± 24.30)%; *df* = 72; *p* < 0.05) in response to ds*ISWI* and ds*BRM* injection, respectively, and were significantly lower than that of the control. The hatching rate of the uninjected females was 77.54 ± 2.25)% (*df* = 72; *p* < 0.05), whilst those of the females injected with water and ds*EGFP* were (76.57 ± 1.80)% (*df* = 72; *p* < 0.05) and (74.45 ± 1.89)% (*df* = 72; *p* < 0.05), respectively (Figure 5C,D).

The lifespans of the females injected with ds*ISWI* (8.21 ± 2.15; *df* = 72; *p* < 0.05) and ds*BRM* (6.95 ± 2.78; *df* = 72; *p* < 0.05) were significantly shorter than that of the uninjected females (18.47 ± 2.17; *df* = 72; *p* < 0.05) and those injected with water (17.63 ± 1.74; *df* = 72; *p* < 0.05) and ds*EGFP* (18.58 ± 2.29; *df* = 72; *p* < 0.05) (Figure 5E).

## 3. Discussion

To the best of our knowledge, the present study is the first to report the full-length cDNA sequence of the chromatin-remodelling ATPase gene *ISWI* in the South American tomato leaf miner *Tuta absoluta*. Bioinformatics analysis disclosed that TaISWI had the same structural domains as the ISWI proteins previously identified in other insect species. It comprised a conserved ATPase domain in the *N*-terminal that hydrolyses ATP and releases energy, and a tandem HAND-SANT-SLIDE domain in the *C*-terminal. Together, they form a nucleosome recognition module that binds an unmodified histone tail and DNA (Figure 2A) [39,40,41]. Prior research on the ISWI protein functional domains showed that efficient remodelling depends on the presence of the *C*-terminal HAND-SANT-SLIDE domain [42,43]. The *C*-terminal of the TaISWI protein shared 89.07% sequence similarity with the ISWI proteins of other insect species. Hence, the function of the chromatin-remodelling ATPase ISWI is relatively conserved among various insect species. We also obtained the partial sequence of the *T. absoluta* chromatin remodelling ATPase *BRM* gene. An earlier study showed that the Bromo domain always resides in the *C*-terminal region of SWI/SNF family remodeller ATPases [34]. This finding was consistent with the *TaBRM* sequence cloned in the present study (Figure 2B).

In numerous insect species, chromatin-remodelling ATPase genes are expressed at different developmental stages. Low *BRM* expression levels were detected in *Drosophila* larvae, pupae, and adult females [44,45]. *BRM* was expressed at all developmental stages of *D. v. virgifera* and *C. lectularius* but reached maxima in eggs and adult females [35,37]. Our quantitative analyses revealed that *TaISWI* and *TaBRM* were also expressed at all developmental stages of *T. absoluta*. However, these genes were downregulated in the second to fourth instars and upregulated in the eggs, pupae, and adult females (Figure 2). Therefore, chromatin-remodelling ATPase genes may regulate embryonic development and reproduction in *T. absoluta* and other insect species.

RNAi is a powerful tool for efficient gene function identification [46] and mRNA-level gene knockdown [47]. Here, we used the RNAi method to assess the functions of two chromatin-remodelling ATPase genes in the South American tomato leaf miner *Tuta* *absoluta*. Relative to the control, *T. absoluta* injected with *TaISWI* or *TaBRM* dsRNA presented with significantly reduced ovarian development, fecundity, hatchability, and longevity at the female adult stage (Figure 4 and Figure 5). Bed bugs (*Cimex* *lectularius*) injected with *BRM* or *ISWI* dsRNA exhibited significantly lower fecundity and hatchability and significantly higher mortality than the control [37,38]. After *BRM* knockdown, the western corn rootworm (*Diabrotica* *virgifera virgifera*) laid normal eggs with very low hatching rates [35]. However, *BRM* knockdown fully suppressed egg formation and caused high mortality in the Neotropical brown stink bug (*Euschistus* *heros*) [36]. *BRM* silencing was lethal to *Drosophila* and fully inhibited oogenesis during its early developmental stages [48,49]. The foregoing studies demonstrated that the chromatin-remodelling ATPases *ISWI* and *BRM* are putative RNAi targets for the control of *T. absoluta* and other insect pests.

The present study also showed distinct differences between female and male *T. absoluta* in terms of *TaBRM* expression. Previous studies reported that differences in gene expression contributed to morphological, behavioural, and physiological distinctions between sexes [50,51]. However, the effects of the differences in *TaBRM* expression between female and male *T. absoluta* in terms of their behaviour and physiology are unknown and merit further investigation by RNAi or CRISPR/Cas9 methods.

## 4. Materials and Methods

### 4.1. Insect Rearing

The tomato leaf miner (*Tuta absoluta*) colony used in the present study was collected in Yuxi, Yunnan Province, in August 2018, and reared on healthy tomato (*Lycopersicon esculentum* Mill, Maofen) plants raised under greenhouse conditions. The insects were maintained in cages in an insectary at 25 ± 2 °C, 50–60% RH, and a 14 h/10 h light/dark cycle. The host plants were individually grown in pots 9 cm in diameter and under the same conditions as the insects.

### 4.2. RNA Isolation and cDNA Synthesis

Total RNA was isolated from the insects with TRIzol Reagent (Invitrogen, Carlsbad, CA, USA) according to the manufacturer’s instructions. RNA concentration and quality were evaluated with a Nano Photometer P330 (Implen, Munich, Germany) and by 1% agarose gel electrophoresis. First-strand complementary DNA (cDNA) derived from the various RNA samples was synthesised with One-Step gDNA Removal and cDNA Synthesis Super Mix Kits (TransGen, Beijing, China).

### 4.3. Chromatin-Remodelling Gene Cloning

The homologous chromatin-remodelling ATPase genes *ISWI* and *BRM* from *Bombyx mori* (XP_012547334.1 and XP_037871728.1) and *Drosophila melanogaster* (NP_523719.1 and NP_536745.4) were used in blastp and tblastn to query *T. absoluta* transcriptome datasets. Specific PCR primers were designed on the basis of the foregoing fragments (Table 1). PCR amplifications were conducted using FastPfu DNA Polymerase (TransGen, Beijing, China). The amplified fragments were purified with an AxyPrepTM DNA Gel Extraction Kit (Axygen, West Orange, NJ, USA) according to the manufacturer’s instructions, cloned into a pEASY-Blunt Vector (TransGen, Beijing, China), and sequenced.

### 4.4. Gene Sequences Analysis and Phylogenetic Relationships

Sequence similarities were analysed with DNAMAN v. 7.0 (Lynnon BioSoft, San Ramon, CA, USA). The open reading frames (ORF) of the cloned chromatin-remodelling ATPase genes were predicted with ORF finder (http://www.ncbi.nlm.nih.gov/orffinder/ (accessed on 17 December 2021)). The molecular weights and isoelectric points were predicted with ExPASy (http://web.expasy.org/protparam/ (accessed on 17 December 2021)). The conserved functional domains were analysed with the online NCBI Conserved Domains Database (https://www.ncbi.nlm.nih.gov/Structure/cdd/wrpsb.cgi (accessed on 17 December 2021)). The 3D structures of the protein domains were plotted with I-TASSER (https://zhanglab.ccmb.med.umich.edu/I-TASSER/ (accessed on 17 December 2021)). Homologous amino acid sequences from other insect species were downloaded from the GenBank database (https://www.ncbi.nlm.nih.gov/genbank/ (accessed on 17 December 2021)) and used in the phylogenetic analyses. Multiple protein sequence alignments were performed with ClustalW (https://www.genome.jp/tools-bin/clustalw (accessed on 18 December 2021)). The phylogenetic tree was plotted by the maximum likelihood (ML) method based on the Whelan Goldman (WAG) model with 1000 bootstrap replications in MEGA7.0 (https://www.megasoftware.net/older_versions (accessed on 18 December 2021)) [52,53].

### 4.5. RT-qPCR

Total RNA extraction and cDNA synthesis were conducted as previously described. RT-qPCR was performed with a Hieff qPCR SYBR Green Master Mix (Yeasen, Shanghai, China) on an ABI 7500 Real-Time PCR System (Applied Biosystems, Foster City, CA, USA). The primers used in the present study are listed in Table 1 and include RpL5 (large subunit 5 ribosomal protein) as an internal control gene to normalise mRNA expression [54,55]. Each PCR reaction was performed in 20µL volumes consisting of 10.0 µL SYBR Green Master Mix, 1.0 µL cDNA template, 0.4 µL of each primer (10 µM), and 8.2 µL ddH_2_O. The cycling procedure was as follows: 95 °C for 5 min, 40 cycles of 95 °C for 10 s and 60 °C for 30 s, and a melting curve analysis. The different development stages of *T. absoluta* included eggs (*n* = 50), instar 1 (*n* = 15), instar 2 (*n* = 6), instar 3 and 4 (*n* = 2), early to late pupae (*n* = 2), newly emerged to mature females and males (*n* = 2). There were three biological replicates, of which each was assessed in triplicate (technical replicates) to ensure reliability. Relative mRNA expression was calculated by the 2^−∆∆Ct^ method [56,57].

### 4.6. Double-Stranded RNA (dsRNA) Synthesis

The dsRNA used in the present study was synthesised with a MEGA script T7 High Yield Transcription Kit (Ambion, Austin, TX, USA) and gene-specific primers containing a T7 promoter sequence (Table 1). The dsRNA quality and concentration were determined by running the sample on 1.0% agarose gel and measuring the absorbance in a Nano Photometer (Implen, Munich, Germany). The concentration was adjusted to 6000 ng/µL for all synthesised dsRNAs to ensure consistent injection volumes among treatments. The dsRNA was stored at −80 °C until use.

### 4.7. dsRNA Injection and Detection

Pre-emergent late female pupae were collected, and 0.6 μg dsRNA was injected into their conjunctiva with a Nanoject III Microinjection System (Drummond, Broomall, PA, USA) under a stereomicroscope (Stemi508; Carl Zeiss AG, Jena, Germany). The controls consisted of uninjected female pupae and those injected with 0.1 µL water or *EGFP* dsRNA. After injection, the female pupae were placed separately in perforated 1.5 mL centrifuge tubes. Upon emergence, the adults were paired with freshly emerged wild male adults in separate plastic bottles containing fresh tomato leaves. Each treatment consisted of 19 biological replicates, and each replicate consisted of 1 female and 1 male. To establish the effects of RNAi on the insects, total RNA was extracted from the female adults at 72 h after dsRNA injection, and the mRNA levels were quantified.

### 4.8. Post-RNAi Ovary Development and Fecundity

Each treated *T. absoluta* was observed, and its ovary development was examined at 4 d post-emergence. The adult females were anaesthetised with ice and their heads, legs, and wings were excised with fine tweezers. The remaining tissues were rinsed twice with 1× phosphate-buffered saline (PBS) twice for 1 min per rinse, washed in 75% (*v*/*v*) alcohol for 1 min, and rinsed twice with sterile water for 2 min per rinse. The ovaries were excised and photographed under an Olympus stereomicroscope (SZX16; Olympus, Tokyo, Japan). The eggs from each pair of adult *T. absoluta* in the control and treatment groups were collected and counted daily. Hatching rates and female longevity were also calculated. There were 19 biological repetitions per treatment.

### 4.9. Statistical Analysis

Statistical analyses were performed in SAS v. 9.4 (SAS Institute, Inc., Cary, NC, USA). Figures were plotted with GraphPad Prism v. 5.0 (GraphPad Software, La Jolla, CA, USA). One-way ANOVA and LSD tests were used to compare differences between control and treatment means. Data are means ± SEM. Differences between treatment means were considered significant at *p* < 0.05.

## 5. Conclusions

The present study performed molecular characterisations and analysed the expression patterns of the chromatin-remodelling ATPases *ISWI* and *BRM* in the destructive South American tomato leaf miner *Tuta absoluta*. Using RNAi, we successfully elucidated the roles of the foregoing genes in female *T. absoluta* fecundity. Injection with *TaISWI* or *TaBRM* dsRNA significantly inhibited ovary development, fecundity, hatchability, and longevity in the adult females. To the best our knowledge, the present study is the first to demonstrate the robust parental RNAi properties contributing to the suppression of *T. absoluta* populations. Moreover, it confirmed that chromatin-remodelling ATPases are promising potential RNAi targets for the control of *T. absoluta* and other insect pests.

## Figures and Tables

**Table 1:** Primers used for cDNA cloning, qPCR, and dsRNA synthesis.

Gene Name	Primer Name	Primer Sequence (5′ to 3′)
Primers for cDNA cloning		
BRM	BRM-F1	AACCATCACGCTAACGC
	BRM-R1	GCTGGAACATGAAGGGAT
	BRM-F2	ATCATCAAGTGCGACAT
	BRM-R2	CCGACAGGACTCTACCG
	BRM-F3	CGGAAGCCAACCTACT
	BRM-R3	CCTGCCCTTTCACTAA
ISWI	ISWI-F1	GCTAATGTATTTGGATAATTT
	ISWI-R1	AGTATGGCGAGTTTCCC
	ISWI-F2	CGTCTCAAATCTGAAGTAG
	ISWI-R2	CCCAATGTCTTTCTGTAGT
	ISWI-F3	CAGAAGTTGGAGAGCCTA
	ISWI-R3	TCTACCGCTCATAACCG
Primers for qPCR		
BRM	BRM-QF	CAACGGAAAACTCAAGGAATACCA
	BRM-QR	GCACCCAGTTTGATAGCGTACTG
ISWI	ISWI-QF	GCTGATGAGATGGGTCTGGGT
	ISWI-QR	CACACTGCTCTTAGGGAAGGACA
RpL5	RpL5-QF	CAGTCGTCGAGCCAGCAACA
	RpL5-QR	TCCCGCATTGAAGGAGACCA
Primers for dsRNA synthesis		
BRM	BRM-DF	TAATACGACTCACTATAGGGGAACCATCACGCTAACG
	BRM-DR	TAATACGACTCACTATAGGGGGATTCGTGGACCGTA
ISWI	ISWI-DF	TAATACGACTCACTATAGGGTATGGATGTAGGGGACG
	ISWI-DR	TAATACGACTCACTATAGGGCCAGTTGGTTAGGGTTG

## Data Availability

Not applicable.

## Supplementary Materials

The following supporting information can be downloaded at: https://www.mdpi.com/article/10.3390/ijms23063267/s1.
