# Supplementary material for: Chromatin-Remodelling ATPases ISWI and BRM Are Essential for Reproduction in the Destructive Pest Tuta absoluta
Source: Int J Mol Sci. 2022 Mar 17;23(6):3267. doi: 10.3390/ijms23063267 (PMC8951242; doi:10.3390/ijms23063267)
Supplement: Supplementary file 1 [file ijms-23-03267-s001.zip › ijms-1611673-supplementary materials.pdf]

A

1	GCTAATGTATTTGGATAATTTATTGTGAATCGTAATAATATAACAGAAGATTTCACAATTAACAATATGTCACAACCTGATGAAGCTATGGATGTAGGGGACGTGGAAGAAAACTCTAAT
1	M S Q P D E A M D V G D V E E N S N
121	GAGTCGTCCAGCGATACCACGTCCTCAAAGGGTAAAGAAGGAGACTTCGAAAGTAAAATTGAGACCGATCGTTCCAAGCGTTTTGATTTTTTGTTGAAACAAACTGAAATATTCTCGCAT
19	E S S S D T T S S K G K E G D F E S K I E T D R S K R F D F L L K Q T E I F S H
241	TTCATGACCAATGCGCCTAAAACCAGTAGTCCTCCTAAGGCTAAAGCCGGTCGACCTAAAAAGGTGAAAGAAGATCTGCCTGAGCAGTCTGAGGATGCTTCTGCAGCAGACCATCGGCAC
59	F M T N A P K T S S P P K A K A G R P K K V K E D L P E Q S E D A S A A D H R H
361	CGCAAGACTGAACAGGAAGAAGATGAGGAACTCCTTGCTGAGACAAACGCTAAGATTAAGCCAATATTTCGCTTTGAAGCATCACCTCCTTACATCAAGAATGGAGAAATGAGAGATTAT
99	R K T E Q E E D E E L L A E T N A K I K P I F R F E A S P P Y I K N G E M R D Y
481	CAAGTGAGAGGTCTCAACTGGATGATATCTCTCTATGAGAATGGTATTAATGGTATCTTGGCTGATGAGATGGGTCTGGGTAAAACATTACAAACCATTTCTCTTCTTGGCTACATGAAA
139	Q V R G L N W M I S L Y E N G I N G I L A D E M G L G K T L Q T I S L L G Y M K
601	AATTTCAGAAATATCCCTGGTCCACACATAGTAATTGTGCCAAAATCAACCCTAACCAACTGGATGAATGAGTTCAAAAAATGGTGTCCTTCCCTAAGAGCAGTGTGTCTTATTGGAGAT
179	N F R N I P G P H I V I V P K S T L T N W M N E F K K W C P S L R A V C L I G D
721	CAGGAAACAAGGAACACATTCATCCGTGAAGTACTGATGCCTGGCAACTGGGATGTGTGCATCACCTCTTACGAGATGATTATCCGTGAGAGATCAGTATTTAAGAAGTTCACTTGGCGC
219	Q E T R N T F I R E V L M P G N W D V C I T S Y E M I I R E R S V F K K F T W R
841	TACATGGTGATTGACGAAGCCCATCGAATCAAGAATGAGAAGTCAAAGTTGTCAGAGCTTCTCCGGGAGTTCAAGAGTATGAACCGGCTGCTGCTCACTGGCACACCGTTGCAAAACAAT
259	Y M V I D E A H R I K N E K S K L S E L L R E F K S M N R L L L T G T P L Q N N
961	CTTCACGAGCTGTGGGCACTTCTTAACTTCTTGTTACCAGATGTATTCAACAGCTCTGATGATTTTGACTCTTGGTTCAACACCAATGCAGCTCTCGGTGATAATCAGCTGGTGTCCCGG
299	L H E L W A L L N F L L P D V F N S S D D F D S W F N T N A A L G D N Q L V S R
1081	TTGCATGCTGTGTTGAGACCGTTCTTACTCAGACGTCTCAAATCTGAAGTAGAAAAGAAATTGAAACCCAAAAAGGAACTCAAAGTTTATGTTGGATTGAGTAAGATGCAAAGGGAGTGG
339	L H A V L R P F L L R R L K S E V E K K L K P K K E L K V Y V G L S K M Q R E W
1201	TATACTAAAGTACTCATGAAGGATATTGATATTGTAAACGGCGCGGGCAAAGTCGAAAAGATGCGTCTCCAGAACATCCTGATGCAGCTCCGCAAGTGCTGCAACCACCCGTACCTGTTC
379	Y T K V L M K D I D I V N G A G K V E K M R L Q N I L M Q L R K C C N H P Y L F
1321	GACGGCGCGGAGCCCGGCCCGCCCTACACCACGGACGAGCATCTCGTCTACAACTGCGGGAAACTCGCCATACTCGACAAACTGCTGCCCAAGCTGCAGCAGCAGGACTCCAGGGTGCTC
419	D G A E P G P P Y T T D E H L V Y N C G K L A I L D K L L P K L Q Q Q D S R V L
1441	ATCTTCTCGCAGATGACCAGGATGCTGGATATTCTGGAGGATTATTGCCTGTGGAGGCAGTACAAGTACTGCCGCTTAGACGGTCAGACCCCACATGAAGACCGCAACCGACAGATCGAG
459	I F S Q M T R M L D I L E D Y C L W R Q Y K Y C R L D G Q T P H E D R N R Q I E
1561	GAGTATAACGCGGAAGGCTCCGAGAAGTTCGTGTTCATGTTGTCCACCCGCGCCGGCGGGCTGGGGATCAACCTCACCTCGGCAGATGTCGTCATCATATACGACTCCGACTGGAACCCG
499	E Y N A E G S E K F V F M L S T R A G G L G I N L T S A D V V I I Y D S D W N P
1681	CAGATGGACCTGCAGGCGATGGACCGCGCGCATCGTATTGGGCAGATGAAACAAGTGCGCGTATTCAGACTGATCACGGAGAACACGGTGGAAGAGAAGATAGTGGAGCGCGCGGAAGTG
539	Q M D L Q A M D R A H R I G Q M K Q V R V F R L I T E N T V E E K I V E R A E V
1801	AAACTACGCCTAGACAAGCTCGTAATCCAGTCCGGCCGGCTCGTGGACCAGAAGAACCAGCTCAACAAGGACGAAATGCTCAACATGATCAGGCACGGGGCCAACCACGTCTTCTCCTCG
579	K L R L D K L V I Q S G R L V D Q K N Q L N K D E M L N M I R H G A N H V F S S
1921	AAGGACTCCGAGATCACCGATGAGGATATTGACTCTATTCTAGCCAAAGGAGAGACCAAGACAGAAGAATTGAAACAGAAGTTGGAGAGCCTGGAGGAGTCATCTCTGAGAGCGTTCTCG
619	K D S E I T D E D I D S I L A K G E T K T E E L K Q K L E S L E E S S L R A F S
2041	ATGGACACCCCCGGCGCGACCGACTCCGTCTACCAATTCGAAGGCGAGGACTACCGGGAGAAGCAGAAGTCGCACCCGCTGGGCAGCTGGATCGAGCCGCCGAAGCGCGAGCGCAAGGCC
659	M D T P G A T D S V Y Q F E G E D Y R E K Q K S H P L G S W I E P P K R E R K A
2161	AACTACGCGGTGGACGCGTACTTCCGCGAGGCGCTGCGCGTCTCCGAGCCCAAGGCGCCGAAAGTGCAGGCGCCGCGCCCCCCCAAGCAGCCCATCGTGCAGGACTTCCAGTTCTTCCCG
699	N Y A V D A Y F R E A L R V S E P K A P K V Q A P R P P K Q P I V Q D F Q F F P
2281	CCGAGGCTGTTCGAGCTGCTGGACCAGGAGATCTACCACTACAGAAAGACATTGGGGTACAAAGTGCCGCGTAACCCGGAGCTGGGCCCCGACGCGGCCAAGATACAGAGAGAGGAGCAG
739	P R L F E L L D Q E I Y H Y R K T L G Y K V P R N P E L G P D A A K I Q R E E Q
2401	AGGAAGATCGACGACGCCGAAGCGCTCACCGAGGAGGAGGTTGCTGAGAAAGAAAACCTTCTCACTCAGGGTTTCACAAACTGGACAAAACGCGACTTCAACCAGTTCATCAAAGCTAAT
779	R K I D D A E A L T E E E V A E K E N L L T Q G F T N W T K R D F N Q F I K A N
2521	GAGAAGTATGGAAGGGATGACATCGAAAATATTGCAAAAGATGTCGAAGGAAAAACGCCCGAAGAGGTGATGGAATACTCGGCAGTGTTCTGGGAGCGCTGCCACGAGCTGCAGGACGTG
819	E K Y G R D D I E N I A K D V E G K T P E E V M E Y S A V F W E R C H E L Q D V
2641	GACCGGATCATGGGGCAGATCGAGCGCGGCGAGGCCAAGATACAGCGCCGCGCCTCCATCAAGAAGGCGCTCGACGCCAAGATGGCGCGCTACCGGGCGCCCTTCCACCAGCTCAGGATA
859	D R I M G Q I E R G E A K I Q R R A S I K K A L D A K M A R Y R A P F H Q L R I
2761	TCCTACGGGACTAACAAGGGGAAGAACTATGTCGAAGAAGAAGACAGGTTCCTGGTGTGCATGCTGCACAAGCTGGGCTTCGACAAGGAGAACGTGTACGAGGAGCTGCGCGCCGCCGTG
899	S Y G T N K G K N Y V E E E D R F L V C M L H K L G F D K E N V Y E E L R A A V
2881	CACGCCGCGCCGCAGTTCCGCTTCGACTGGTTCCTCAAGTCGCGCACGGCCGTGGAGCTGCAGCGCAGATGCAACACATTGATCACACTCATCGAAAGAGAGAACCAAGAGCTCGAAGAG
939	H A A P Q F R F D W F L K S R T A V E L Q R R C N T L I T L I E R E N Q E L E E
3001	AAGGAGCGCGCCGAGAAGAAAAAGAAGAGCGGCAGCGCCAACCAGAACACGCCCGGCGCCGCCAGCAAGGGCGCCGGCGCCGGCAAGCGCAAGGCAGACGCCGCGCAGGACTCCGCGCAG
979	K E R A E K K K K S G S A N Q N T P G A A S K G A G A G K R K A D A A Q D S A Q
3121	AAACAGAAGAAGAAGAAGAAATGAAACCCCGCCGTCCGGGCGACTGCAGCCCCCGCCCTGGCGCGCGCCACCAGGGCCTCCGCCCGCCAGTGACGCCCGCCACTACCATCAGCTCTACAT
1019	K Q K K K K K
3241	ACGAGGCTGACAGCGGATTTTTGATTATTCCAAAATAAAAGTTCCACATCTTAACAGCATATAAATTCGTCCTGTCTTTTTTTATCTCTCAGAACCGCCCCGTGCCAGAGCGAGAAAGGC
3361	GTACTCGGCTGAACTGTTGAGGCGATCCCACTCCCTTGCTGAACCATATGGCGGTTATGAGCGGTAGA

B

```
1      AACCATCACGCTAACGCCGAGCGCGAGCAGAAGAAGGAACAGGAGCGTATCGAGAAGGAACGTATGCGCCGCCTGATGGCGGAGGACGAGGAGGGTTACCGGAAGCTCATCGACCAGAAG
1                                                                    M  R  R  L  M  A  E  D  E  E  G  Y  R  K  L  I  D  Q  K
121    AAGGACAAGCGGCTGGCGTTCCTGTTGTCGCAGACGGACGAGTACATCGCCAGCCTCACCGAGATGGTGAAGCAGCACAAACAGGAGCAGCGCAAGAAACAAGTCGAGGAGGAGAAACGG
20      K  D  K  R  L  A  F  L  L  S  Q  T  D  E  Y  I  A  S  L  T  E  M  V  K  Q  H  K  Q  E  Q  R  K  K  Q  V  E  E  E  K  R
241    AAGCGGAAATCCCGGAAGAAGAAGTTGCTGGAGGGCGGGGAGATCGACGCGATGGACGACAGCTCGCAGACCTCCGACTCGCGTGTCACCGTCATGGACCCCAAGACGGGCGAAGTGTTG
60      K  R  K  S  R  K  K  K  L  L  E  G  G  E  I  D  A  M  D  D  S  S  Q  T  S  D  S  R  V  T  V  M  D  P  K  T  G  E  V  L
361    AAGGGCGAGGAAGCCCCGCTGCTGTCTCAGCTGAAGGGCTGGATGGAGCAGCACCCCGGCTGGGAGGTGGTGTCGGACTCCGACGACTCGGGCGACGACAGCCAGGACGACGACGGACAC
100     K  G  E  E  A  P  L  L  S  Q  L  K  G  W  M  E  Q  H  P  G  W  E  V  V  S  D  S  D  D  S  G  D  D  S  Q  D  D  D  G  H
481    TACAAGAGACACAAACACAGGGACGACAAGAATGAGAACAGGGAGAAGACCGAGGAGGAGAAGGCGCGCGAGATGATCAAGAAAGCCAAGGTGGAGGACGACGAGTACAAGACAGAGGAG
140     Y  K  R  H  K  H  R  D  D  K  N  E  N  R  E  K  T  E  E  E  K  A  R  E  M  I  K  K  A  K  V  E  D  D  E  Y  K  T  E  E
601    CAGACATACTACAGCATTGCTCATACGGTCCACGAATCCGTCACAGAACAAGCCAGCATTTTGGTCAACGGAAAACTCAAGGAATACCAGATCAAGGGTCTGGAATGGCTGGTGTCTTTG
180     Q  T  Y  Y  S  I  A  H  T  V  H  E  S  V  T  E  Q  A  S  I  L  V  N  G  K  L  K  E  Y  Q  I  K  G  L  E  W  L  V  S  L
721    TTCAACAACAACCTGAACGGTATCCTGGCGGACGAGATGGGTCTCGGCAAGACCATCCAGACGATAGCGCTGGTCACATACCTCATGGAGAAGAAGAAAGTCAACGGACCATTCCTCATC
220     F  N  N  N  L  N  G  I  L  A  D  E  M  G  L  G  K  T  I  Q  T  I  A  L  V  T  Y  L  M  E  K  K  K  V  N  G  P  F  L  I
841    ATTGTACCGCTCAGTACGCTATCAAACTGGGTGCTGGAGTTCGAGAAGTGGGCGCCGACCGTGTGCGTAGTGTCGTACAAGGGCTCGCCCGCGTCGCGCCGCCTCGTGCAGAACCAGATG
260     I  V  P  L  S  T  L  S  N  W  V  L  E  F  E  K  W  A  P  T  V  C  V  V  S  Y  K  G  S  P  A  S  R  R  L  V  Q  N  Q  M
961    AGGTCCACCAAGTTCAACGTGCTGCTCACCACCTACGAGTACGTCATCAAGGACAAGGGAGTGCTCGCTAAGGTGCAATGGAAGTACATGATAATCGACGAGGGCCACCGCATGAAGAAC
300     R  S  T  K  F  N  V  L  L  T  T  Y  E  Y  V  I  K  D  K  G  V  L  A  K  V  Q  W  K  Y  M  I  I  D  E  G  H  R  M  K  N
1081   CACCACTGCAAGCTGACGCAGGTGCTGAACACGCACTACATAGCGCCGCACCGCCTGCTGCTGACCGGCACGCCGCTGCAGAACAAGCTGCCCGAACTCTGGGCGCTGCTGAACTTCCTC
340     H  H  C  K  L  T  Q  V  L  N  T  H  Y  I  A  P  H  R  L  L  L  T  G  T  P  L  Q  N  K  L  P  E  L  W  A  L  L  N  F  L
1201   CTGCCGTCCATATTCAAGAGTTGCTCCACCTTCGAACAGTGGTTCAATGCGCCCTTCGCTACCACTGGAGAAAAGGTGGAACTAAACGAGGAAGAAACGATCCTGATCATCCGTCGTCTA
380     L  P  S  I  F  K  S  C  S  T  F  E  Q  W  F  N  A  P  F  A  T  T  G  E  K  V  E  L  N  E  E  E  T  I  L  I  I  R  R  L
1321   CACAAAGTGCTGCGTCCGTTCTTACTGCGGCGCCTGAAGAAGGAAGTAGAGAGCCAGCTGCCCGACAAAGTGGAGTACATCATCAAGTGCGACATGAGCGGGCTGCAGCGAGTGCTCTAC
420     H  K  V  L  R  P  F  L  L  R  R  L  K  K  E  V  E  S  Q  L  P  D  K  V  E  Y  I  I  K  C  D  M  S  G  L  Q  R  V  L  Y
1441   AAACACATGCAGTCGAAGGGCGTGCTGCTGACGGACGGCTCGGAGAAGGGCACGAAGGGCAAGGGCGGGGCGAAGGCGCTCATGAACACCATCGTGCAGCTGCGCAAGCTCTGCAACCAT
460     K  H  M  Q  S  K  G  V  L  L  T  D  G  S  E  K  G  T  K  G  K  G  G  A  K  A  L  M  N  T  I  V  Q  L  R  K  L  C  N  H
1561   CCCTTCATGTTCCAGCACATCGAGGAGAAGTTCTGCGACCACGTCGGCTCTGGCAGCACCGTCGTTTCTGGTCCGGACCTGTACCGCGTGTCGGGCAAGTTCGAGCTGCTGGACCGCATC
500     P  F  M  F  Q  H  I  E  E  K  F  C  D  H  V  G  S  G  S  T  V  V  S  G  P  D  L  Y  R  V  S  G  K  F  E  L  L  D  R  I
1681   CTGCCCAAGCTGAAGCGCACCGGCCACCGCGTGCTCGTGTTCTGCCAGATGACGCAGTGCATGACCATCATCGAGGACTACCTCTCCTGGAGGGGCTTCCAATACTTGCGGCTGGACGGT
540     L  P  K  L  K  R  T  G  H  R  V  L  V  F  C  Q  M  T  Q  C  M  T  I  I  E  D  Y  L  S  W  R  G  F  Q  Y  L  R  L  D  G
1801   ATGACGAAGGCGGAGGACCGTGGCGAGCTGCTCAAGAAGTTCAACTGCAAGGACTCGGAATATTTCCTCTTCTTGCTGTCTACGAGAGCTGGTGGACTTGGACTCAATCTCCAGTCGGCT
580     M  T  K  A  E  D  R  G  E  L  L  K  K  F  N  C  K  D  S  E  Y  F  L  F  L  L  S  T  R  A  G  G  L  G  L  N  L  Q  S  A
1921   GACACTGTCATCATCTTCGATTCCGATTGGAATCCGCATCAGGATCTGCAAGCGCAAGACCGCGCGCATCGTATCGGACAACGTAACGAAGTGCGCGTGTTGCGACTGATGACCGTCAAC
620     D  T  V  I  I  F  D  S  D  W  N  P  H  Q  D  L  Q  A  Q  D  R  A  H  R  I  G  Q  R  N  E  V  R  V  L  R  L  M  T  V  N
2041   TCCGTCGAGGAACGGATTCTTGCCGCCGCCAGGTACAAATTGAACATGGACGAGAAGGTTATCCAAGCCGGTATGTTCGATCAAAAGTCCACCGGCTCAGAGCGACAGCAGTTCCTGCAG
660     S  V  E  E  R  I  L  A  A  A  R  Y  K  L  N  M  D  E  K  V  I  Q  A  G  M  F  D  Q  K  S  T  G  S  E  R  Q  Q  F  L  Q
2161   AGCATTCTGCATCAAGACGGCGACGATGAAGAAGAAGAAAATGAACTCCCGGACGACGACCTCATCAACGAGATGATCGCGCGCTCGGAGGAGGAGCTCGAGATCTTCAAACAGATCGAC
700     S  I  L  H  Q  D  G  D  D  E  E  E  E  N  E  L  P  D  D  D  L  I  N  E  M  I  A  R  S  E  E  E  L  E  I  F  K  Q  I  D
2281   ATCGAGCGGAAGAAAACCGAAACCACCTCCCGTCTCATAGAGGAGTGCGAGCTGCCCGACTGGCTCACCAAGAACGAGGACGAAGTTGTGTGCAACAAGGGCCAAGGGTGGGGCAACTTC
740     I  E  R  K  K  T  E  T  T  S  R  L  I  E  E  C  E  L  P  D  W  L  T  K  N  E  D  E  V  V  C  N  K  G  Q  G  W  G  N  F
2401   CTGGACGCTGAGGAGACGTTCGGGCGCGGCTCCCGGCAGCGCAAAGAGGTTGACTACACGGACTCGCTCACGGAGAAGGAGTGGCTCAAGGCCATCGACGACGAGTTCGACGACGAGGAG
780     L  D  A  E  E  T  F  G  R  G  S  R  Q  R  K  E  V  D  Y  T  D  S  L  T  E  K  E  W  L  K  A  I  D  D  E  F  D  D  E  E
2521   GAGGAGGACGACGACGACGAAGTGCTCGACAAGAAGCGCAAGAAGGGCCGCAAGCGACGCCGCCAAGAAGAGTCGGATGAGGAAGAAGTGGCCAGCTCATCTAAGAAGAAGAATAAGACG
820     E  E  D  D  D  D  E  V  L  D  K  K  R  K  K  G  R  K  R  R  R  Q  E  E  S  D  E  E  E  V  A  S  S  S  K  K  K  N  K  T
2641   GAAGCCAACCTACTGAAGAAAAGGCTCAAGAGCATTATGAAGAAAGTCGTGGACTACGCTGACGAGGGCGGTAGAGTCCTGTCGGAGCCGTTCATGAAGCTGCCGTCGCGGCGCGAGCTG
860     E  A  N  L  L  K  K  R  L  K  S  I  M  K  K  V  V  D  Y  A  D  E  G  G  R  V  L  S  E  P  F  M  K  L  P  S  R  R  E  L
2761   CCCGACTACTACGACGTCATCAAGAAACCGCTCGATATCAAGAAAATCATGACGCGCATCGAAGACGGGAAGTACAACGACATTACGGACTTGGAGCGCGATTTCTTCACTCTGTGTCAG
900     P  D  Y  Y  D  V  I  K  K  P  L  D  I  K  K  I  M  T  R  I  E  D  G  K  Y  N  D  I  T  D  L  E  R  D  F  F  T  L  C  Q
2881   AACGCACAAACCTACAACGAGGAGGCGTCGCTCATCTACGCGGACTCCGTGCGACTGAGGAATGTTTTCATTGAGTTCCGACGCAACTACGAGGCGGGCAACAATTCCGACAACTCCGAC
940     N  A  Q  T  Y  N  E  E  A  S  L  I  Y  A  D  S  V  R  L  R  N  V  F  I  E  F  R  R  N  Y  E  A  G  N  N  S  D  N  S  D
3001   GATGACAAAGATAAAGAAGAAGAAGACTCGGACGGCGAGTCGAACCGCTCGGTCAAGATGAAGATCAAGCTGAAAGGCAAAGGCAAGGGCACGCCCTCGAGGAAAAAGAAACAAAAGAAG
980     D  D  K  D  K  E  E  E  D  S  D  G  E  S  N  R  S  V  K  M  K  I  K  L  K  G  K  G  K  G  T  P  S  R  K  K  K  Q  K  K
3121   TATATCTCCGACGACGAGGACTATGAGGAGGATTGAATCCGGAGTCCAACATATGTCCCGGGTTGGAATATTTGTCCAAGCCAATATTACAATAAATATGAATATGTCCTGTATTCTGAA
1020    Y  I  S  D  D  E  D  Y  E  E  D
3241   GTGTTAATCCACGTTTTTCTGACTGGACATAATATCTCGTATCTTTTTACTAAGTGCAGCTTTGCTGCCTTGATATACATTCTCATAGTAGTGTCCAACTACTTGTAGAGATCTGGCAAC
3361   CCTGCATCTAATCAACCAAGAGAAAACAACTTTTTTTTTTATAGCTAGACAAGTAATTCGGGGAACAGGCAAGTTCTAGGATTTTAGTGAAAGGGCA
```

Figure S1. (A) Full-length cDNA sequence of *TaISWI* and its deduced amino acid sequence. (B) Partial-length *TaBRM* cDNA sequence and its deduced aa sequence.

A

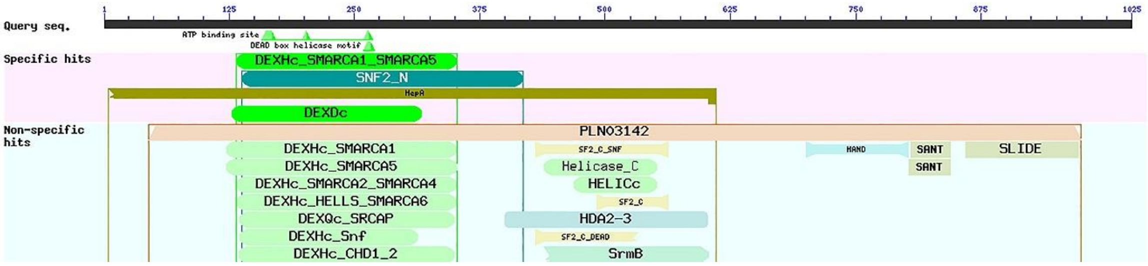

B

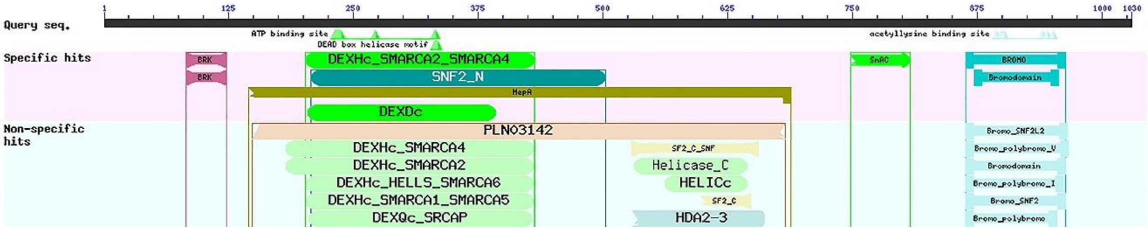

Figure S2. Conserved domain prediction of ISWI (A) and BRM (B) proteins in *Tuta absoluta*. The domains were analysed with the online NCBI Conserved Domains Database (https://www.ncbi.nlm.nih.gov/Structure/cdd/wrpsb.cgi).

A

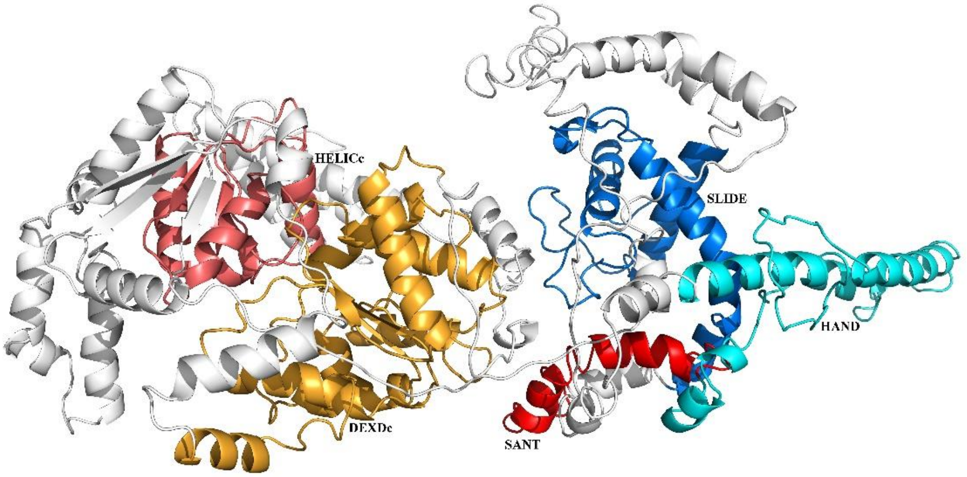

B

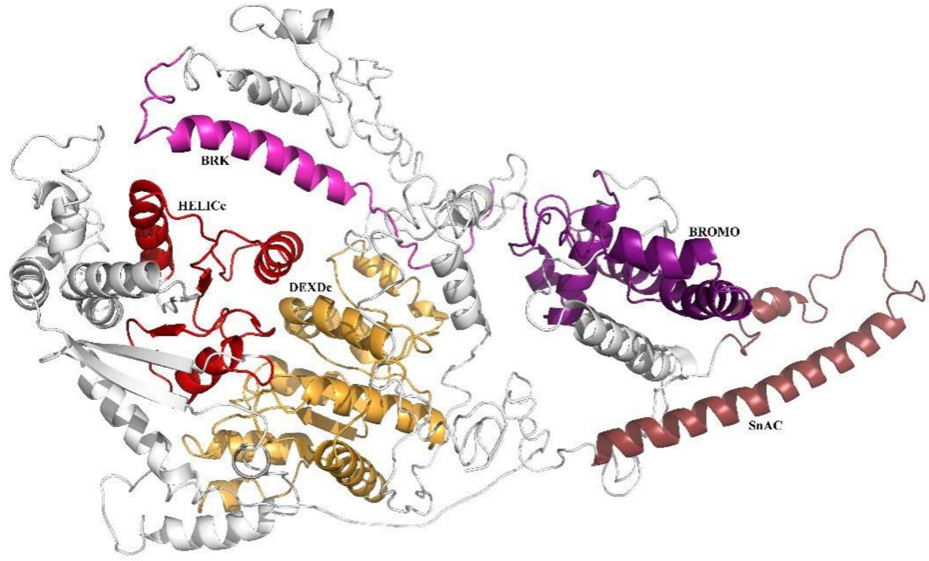

Figure S3. Predicted 3D structure of ISWI (A) and BRM (B) proteins in *Tuta absoluta.*

A

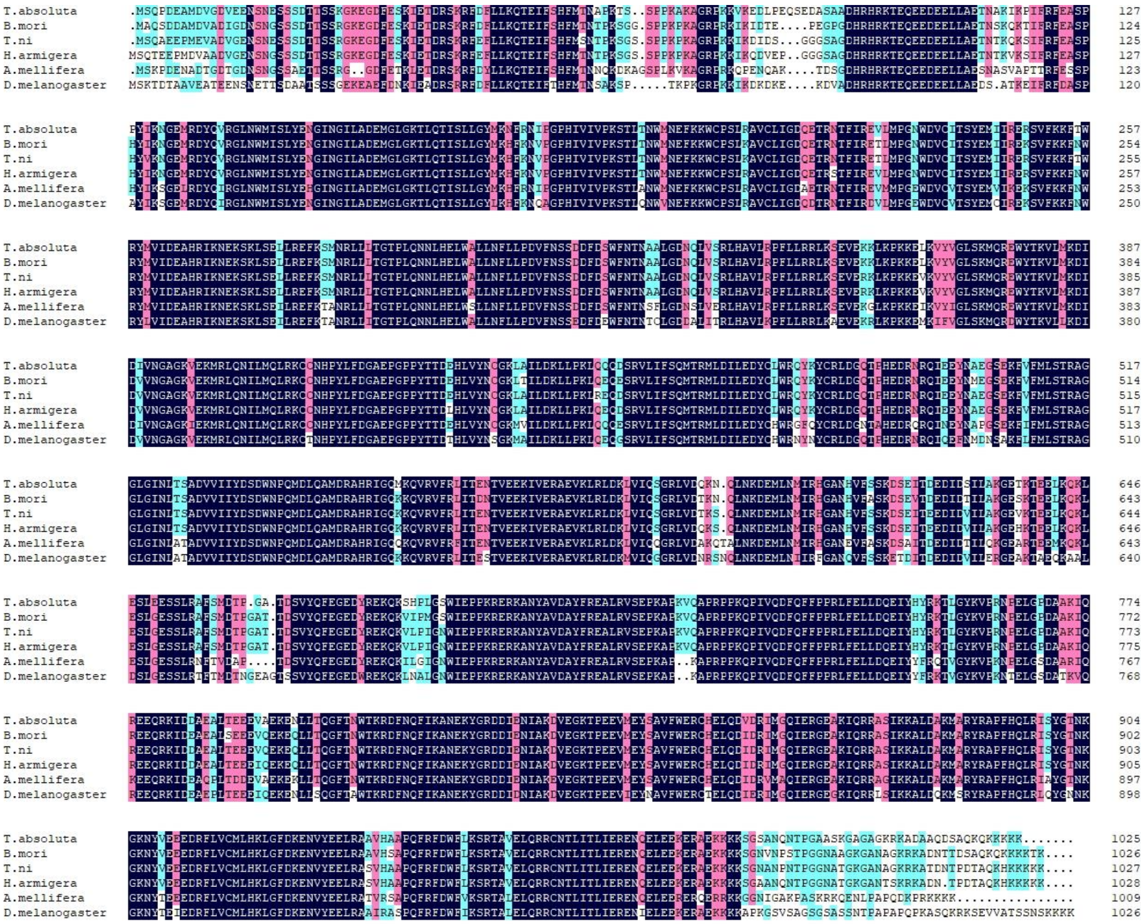

B

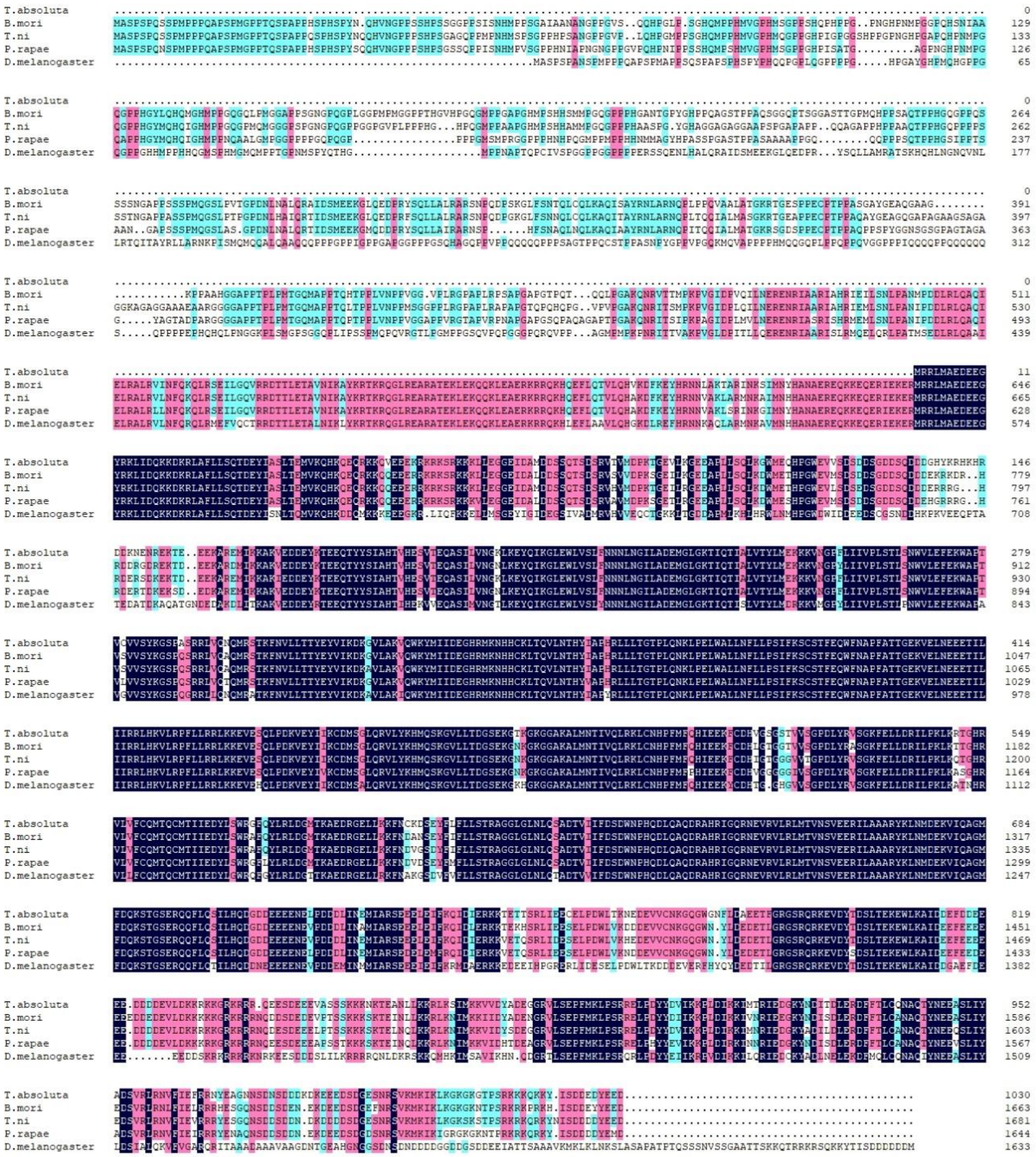

Figure S4. (A) Multiple alignments of the ISWI protein sequences from *Tuta absoluta* and other insects. The results revealed that the deduced protein sequence of *TaISWI* was highly conserved when compared to previously identified ISWI protein sequences. *T. absoluta*: *Tuta absoluta*; *B. mori*: *Bombyx mori* (XP_012547334.1); *T. ni*: *Trichoplusia ni* (XP_026747092.1); *H. armigera*: *Helicoverpa armigera* (XP_021184575.1); *A. mellifera*: *Apis mellifera* (XP_006565266.1); *D. melanogaster*: *Drosophila melanogaster* (NP_523719.1). (B) Multiple alignments of the BRM protein sequences from *Tuta absoluta* and other insects. *B. mori* (XP_037871728.1); *T. ni* (XP_026725796.1); *P. rapae*: *Pieris rapae* (XP_022130779.1); *D. melanogaster* (NP_536745.4).

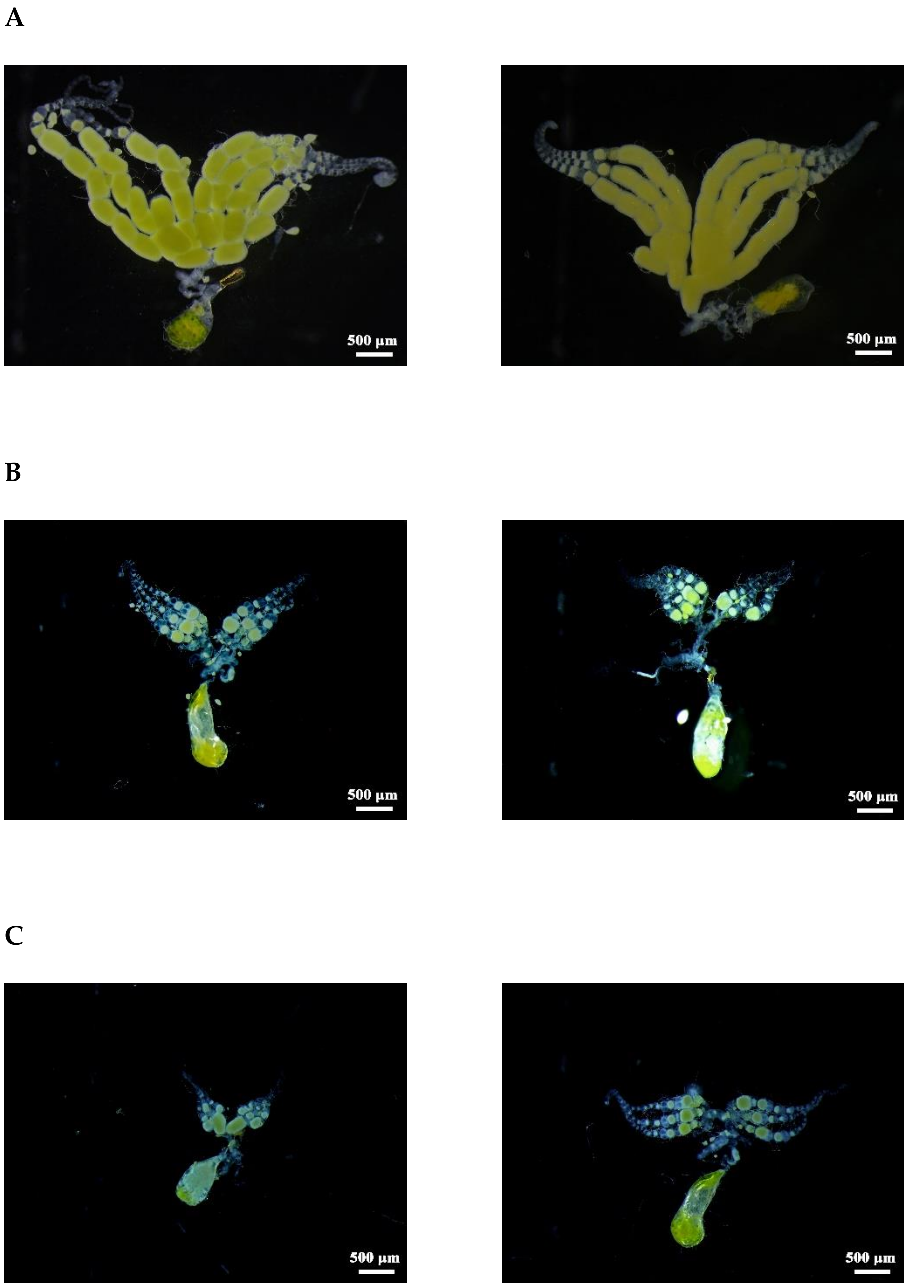

Figure S5. More pictures of the effects of ds*ISWI* and ds*BRM* on *Tuta absoluta* ovary development. (A) Ovaries of uninjected 4-d females. (B) Abnormal ovaries of 4-d females injected with ds*ISWI*. (C) Abnormal ovaries of 4-d females injected with ds*BRM.*

Table S1. Details of protein sequences used for phylogenetic analysis.

ISWI

Insect species	GenBank accession number	Insect species	GenBank accession number
Polistes canadensis	XP_014609868.1	*Ooceraea biroi*	XP_011352072.1
Ceratina calcarata	XP_017890682.1	*Camponotus floridanus*	XP_011254438.1
Linepithema humile	XP_012220642.1	*Bombus impatiens*	XP_024221024.1
Apis mellifera	XP_006565266.1	*Apis florea*	XP_012344097.1
Rhopalosiphum maidis	XP_026823151.1	*Myzus persicae*	XP_022171705.1
Aphis gossypii	XP_027848482.1	*Acyrthosiphon pisum*	XP_001945595.1
Sipha flava	XP_025406176.1	*Diuraphis noxia*	XP_015375778.1
Trichoplusia ni	XP_026747092.1	*Helicoverpa armigera*	XP_021184575.1
Spodoptera litura	XP_022829094.1	*Agrilus planipennis*	XP_025836927.1
Nicrophorus vespilloides	XP_017778534.1	*Bombyx mandarina*	XP_028037570.1
Onthophagus taurus	XP_022908972.1	*Aethina tumida*	XP_019866350.1
Papilio machaon	XP_014355986.1	*Papilio xuthus*	XP_013175309.1
Papilio polytes	XP_013135016.1	*Galleria mellonella*	XP_026765070.1
Bombyx mori	XP_012547334.1	*Bicyclus anynana*	XP_023952043.1
Leptinotarsa decemlineata	XP_023014233.1	*Diabrotica virgifera*	XP_028129819.1
Plutella xylostella	XP_011555157.1	*Ostrinia furnacalis*	XP_028158215.1
Dendroctonus ponderosae	XP_019754255.1	*Bactrocera dorsalis*	XP_011197903.1
Drosophila melanogaster	NP_523719.1	*Ceratitis capitate*	XP_004517436.1
Anoplophora glabripennis	XP_018572974.1	*Bactrocera latifrons*	XP_018803107.1

BRM

Insect species	GenBank accession number	Insect species	GenBank accession number
Bombyx mori	XP_037871728.1	*Pararge aegeria*	XP_039765300.1
Leptinotarsa decemlineata	XP_023029618.1	*Bombyx mandarina*	XP_028033541.1
Hyposmocoma kahamanoa	XP_026323568.1	*Pieris rapae*	XP_022130779.1
Maniola hyperantus	XP_034841481.1	*Ostrinia furnacalis*	XP_028176736.1
Spodoptera litura	XP_022824228.1	*Trichoplusia ni*	XP_026725796.1
Manduca sexta	XP_037300295.1	*Drosophila busckii*	ALC44792.1
Drosophila melanogaster	NP_536745.4	*Bactrocera tryoni*	XP_039964134.1
Cyphomyrmex costatus	XP_018396747.1	*Sipha flava*	XP_025423084.1
Vollenhovia emeryi	XP_011868917.1	*Myzus persicae*	XP_022166801.1
Trachymyrmex septentrionalis	XP_018355410.1	*Bombus terrestris*	XP_012166376.1

Insect species	GenBank accession number	Insect species	GenBank accession number
Camponotus floridanus	XP_011257142.2	*Nylanderia fulva*	XP_029162796.1
Apis mellifera	XP_026300357.1	*Apis florea*	XP_012343937.1
Melanaphis sacchari	XP_025204346.1	*Apis dorsata*	XP_006622867.1
Rhopalosiphum maidis	XP_026814829.1	*Photinus pyralis*	XP_031335174.1
Acyrthosiphon pisum	XP_001947872.2	*Polistes dominula*	XP_015191338.1
Sitophilus oryzae	XP_030758143.1	*Tribolium castaneum*	EEZ97706.2
Anoplophora glabripennis	XP_018562326.2	*Agrilus planipennis*	XP_025830418.1
